# Stock market prediction using Altruistic Dragonfly Algorithm

**DOI:** 10.1371/journal.pone.0282002

**Published:** 2023-04-14

**Authors:** Bitanu Chatterjee, Sayan Acharya, Trinav Bhattacharyya, Seyedali Mirjalili, Ram Sarkar

**Affiliations:** 1 Department of Computer Science and Engineering, Jadavpur University, Kolkata, West Bengal, India; 2 Centre for Artificial Intelligence Research and Optimization, Torrens University, Fortitude Valley, Queensland, Australia; 3 Yonsei Frontier Lab, Yonsei University, Seoul, South Korea; National Institute of Technology, India (Institute of National Importance), INDIA

## Abstract

Stock market prediction is the process of determining the value of a company’s shares and other financial assets in the future. This paper proposes a new model where Altruistic Dragonfly Algorithm (ADA) is combined with Least Squares Support Vector Machine (LS-SVM) for stock market prediction. ADA is a meta-heuristic algorithm which optimizes the parameters of LS-SVM to avoid local minima and overfitting, resulting in better prediction performance. Experiments have been performed on 12 datasets and the obtained results are compared with other popular meta-heuristic algorithms. The results show that the proposed model provides a better predictive ability and demonstrate the effectiveness of ADA in optimizing the parameters of LS-SVM.

## 1 Introduction

Stock market trading involves buying and selling of shares of a company listed on the stock exchange. Investors seek to maximize profits and minimize risks at every turn. Predicting the ever-changing trends of the share market is a difficult task, yet an accurate prediction can lead to huge gains for the investors. With the increase in awareness about the importance of having a diversified portfolio, more and more individuals are looking to invest smartly in the stock market as a part of their personal finance management. As a result, forecasting the stock market has become a major area of study in recent times. Researchers have used data mining and machine learning approaches to predict stock prices. These techniques are preferred over traditional statistical methods as they can uncover hidden patterns in the historical stock data to get an idea about the future price.

Auto Regressive Integrated Moving Average (ARIMA) is a commonly used technique to predict time series data which has a general trend associated with it [1]. However, this is not very suitable for application in stock market data which is full of non-linear and irregular in nature. To counter that many researchers had used Artificial Neural Networks (ANN) in the past [2]. However, overfitting is often a concern for ANNs because of the large number of parameters to tune and limited knowledge about the relevance of the input with respect to the problem at hand. To fix that hybridization with other optimization algorithms have also been tried by many researchers. Another very popular method is the use of Support Vector Machine (SVM), which is a supervised learning technique introduced by Cortes and Vapnik [3] in 1995. It tends to find the globally optimal solution, unlike ANNs which get stuck in local minima [4].

Another advantage of SVMs is that it is computationally faster as its time complexity is quadratic with respect to the training data, whereas logistic regression has cubic time complexity. However, SVMs do not perform very well in the case of high noise and overlapping target classes. SVMs use quadratic programming (QP) with inequality constraints. To simplify the optimization process, Suykens and Vandewalle introduced a modified SVM called Least Squares SVM (LS-SVM) [5], which involves a least squares function with equality constraints to produce a linear system satisfying the Karush-Kuhn-Tucker (KKT) conditions for getting an optimal solution. In this process, choosing optimal values for the regularization and kernel parameters is vital to make the model robust as the parameters have a considerable impact on the classification or regression performance [6]. In this paper, we take a look at how Dragonfly optimization algorithm with altruism is used to select the parameters of SVM which is then used to predict the stock prices.

The COVID-19 pandemic ongoing for the past couple of years has caused a major shock all over the world. The financial markets have also been badly hit because of lockdowns and other measures placed by governments [7, 8]. The economic shock has spread to stock markets as well [9]. After a period of bear markets worldwide [10], stock markets in many countries across the world are experiencing a historic boom and this has led to huge demand for accurate stock market prediction models. This is because people and corporate can then make huge profits by anticipating a rise or fall in stock prices. The proposed work responds to this demand and seeks to develop a reliable model for predicting future stock prices of companies.

The highlighting points of this paper are listed below:

- Application of the concept of altruism to improve the exploitation ability of Dragonfly Algorithm (DA).
- Application of Altruistic Dragonfly Algorithm (ADA) to find out the optimal parameters for LS-SVM in the proposed model.
- Experimentation on twelve stock price datasets to predict the future prices.
- Comparison of the proposed model with several meta-heuristics against datasets of 12 companies.

The rest of the paper is organized as follows: Section 2 provides an in-depth literature review. SVM and DA algorithms are presented in Section 3. Section 4 presents the proposed algorithm. Sections 5 and 6 will provide the results, discussions, and conclusions.

## 2 Literature survey

SVM and ANN are perhaps the most widely used methods for prediction of stock prices [11]. In recent years, ANNs have been extensively used for solving optimization problems. They are generally hybridized with other optimization algorithms like PSO (Particle Swarm Optimizer), GA (Genetic Algorithm), WOA (Whale Optimization Algorithm), etc. They have the advantage of being able to learn complex patterns in the input data as well as effectively increasing the searching capability of the algorithm through the use of the hybridized optimization algorithms. ANN with PSO and Cuckoo search has been used to solve performance problems [12]. ANN with GA has been used for bankruptcy prediction with very good results [13]. GA has been hybridized with ANN to solve the lipase production problem for penicillium [14]. ANN with Spot Welding Optimization to determine aluminium alloy measurement [15]. Apart from these types of hybridizations, Lyapunov based ANNs have also been used extensively. Lyapunov based NN method is used for safe learning of dynamic systems [16]. Adaptive Lyapunov-based neural network was used for sensorless control of permanent magnet synchronous machines [17]. SVM had been previously used to predict stock prices [18] and ANN as well [19]. SVM was later combined with Empirical Mode Decomposition for improved stock market prediction [20]. Apart from SVM, other notable forecasting methods used include Variable Method Decomposition (VMD) combined with Long Short-Term Memory (LSTM) [21], and wavelet kernel support vector [22]. Meta-heuristics became popular in the finance domain and are nowadays being increasingly used for stock market forecasting. Meta-heuristics can be categorized as nature inspired or non-nature inspired. Amongst them, the former category is more popular due to various advantages: easy implementation, modification and enhancement, no complex mathematical derivation, and its ability to not get stuck in a local optima. A very old and time-tested algorithm in this category is Genetic Algorithm (GA) [23]. Further on, nature inspired algorithms are broadly classified into these types: evolutionary, swarm-based, physics-based and human-based algorithms. We now give examples for each of these four types as follows:

- **Evolutionary algorithms**: The mechanism for this type of algorithm is motivated by biological reproduction, crossover, mutation, natural selection and other evolutionary processes. Some popular methods in this category include GA and Genetic Programming [24].
- **Swarm-based algorithms**: These algorithms are based on the behavior of social animals such as nest-building. In swarms, individuals interact with each other and try to improve their quality by utilizing the knowledge gained by the entire swarm. Famous examples are Particle Swarm Optimization (PSO) [25] and Mayfly Algorithm (MA) [26].
- **Physics-based algorithms**: These type of algorithms are based on physical phenomenon or physical-chemical interaction. They may be inspired by physics, chemistry, music, etc. An old and popular algorithm in this class is Simulated Annealing (SA) [27] based on the annealing of metals. Other examples are Gravitational Search Algorithm (GSA) [28] and Harmony Search (HS) Algorithm [29].
- **Human-based algorithms**: They mimic human behavior. An example is League Championship Algorithm (LCA) [30].

**Fig 1:**
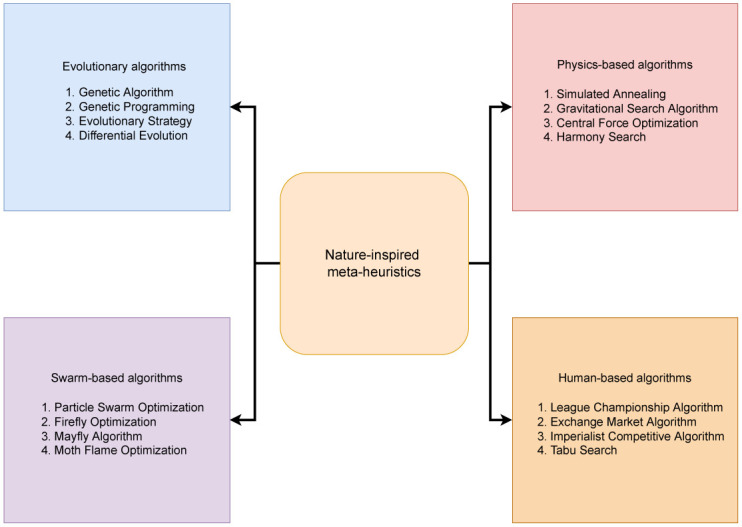
Various categories of nature-inspired meta-heuristic algorithms.

The four types defined above are pictorially shown in Fig 1. Some notable meta-heuristics used for stock market prediction include PSO [31, 32], Artificial Bee Colony [33], Butterfly optimization [34] and GA [35]. Grey Wolf Optimization was combined with Elman Neural Network (ENN) to predict stock prices [36]. A self-adaptable step size optimization algorithm was combined with Fruit Fly Optimization in [37] and used for stock market analysis. The algorithm reduced the step size of fruit flying migration with each iteration, thereby increasing the convergence precision. In [38], PSO was used for the selection of parameters of SVM, which would otherwise be time-intensive and require trial-and-error. However, the high computation time of PSO in determining the parameter values was a significant drawback of this model. The parameters of Convolutional Neural Network (CNN) was improved using GA to forecast stock prices [39]. Here, GA was employed in the search for CNN’s optimal architecture, including kernel size of the convolutional layers, the number of kernels, and the pooling window size, which allows to make use of neural networks’ ability to model complex nonlinear functions while automatically solving the optimization problem. However, it is still difficult to determine a suitable model that can successfully reflect the characteristics of the problem and efficiently learn the data patterns, due to the numerous controlling parameters.

An Online Sequential Extreme Learning Machine (OSELM) model was optimized using Firefly Algorithm (FA) for financial time series forecasting [40]. Hybrid meta-heuristic algorithms are also becoming quite popular nowadays. In this type of method, an algorithm is combined with another of the same or different type. One of the algorithms is generally used for local search, i.e., for enhancing the exploitation power of the model. They usually perform better than the original algorithmic components. Hybrid meta-heuristics used for stock market prediction include PSO combined with Centre of Mass (COM) technique [41], and LS-SVM combined with traditional methods like PSO, Artificial Bee Colony (ABC) algorithm among others [42].

The works described here do not study and predict fluctuating stock data obtained during the COVID-19 outbreak, whereas our proposed work specifically addresses this gap. Moreover, LS-SVM is a much simpler method when compared to other models such as deep learning based models. Hence, its implementation is easier and the model proposed here will enable new researchers to quickly learn and work more in this domain.

## 3 Preliminaries

### 3.1 Least square support vector machine

The conventional SVM is a supervised machine learning model which is constructed with QP [43], where a convex cost function is minimized using inequality constraints. LS-SVM was proposed by Suykens and Vandewalle in [5] as a simpler form of the conventional SVM. Here, the classification or regression problem is solved using a set of linear equations with equality constraints.

Consider X as the input *n* × *m* data matrix and y as the output *n* × 1 output vector. Given the ${\left\{x_{i},y_{i}\right\}}_{i=1}^{n}$ training dataset, where *x*_*i*_ ∈ *R*^*m*^, *y*_*i*_ ∈ *R*, the objective of the LS-SVM is to construct the function *f*(*x*) = *y*, which represents the relation between the input *x*_*i*_ and output *y*_*i*_. The function is as follows:

$$\begin{array}{c} f\left(x\right)=w^{T}\phi \left(x\right)+b \end{array}$$ (1)

where w is the weight vector in the dimension of feature space, *ϕ*(*x*) is a non-linear mapping from the input space to a higher dimensional feature space and *b* ∈ *R* is the bias term. LS-SVM computes Eq 1 from a similar minimization problem found in the SVM method [3]. LS-SVM uses equality constraints based on a least square cost function. The minimization problem and the equality constraints of LS-SVM are defined as follows:

$$\begin{array}{c} Min:\;J_{\left\{w,b,e\right\}}=\frac{1}{2}w^{T}w+\frac{1}{2}\lambda \sum_{k=1}^{n}e_{k}^{2} \end{array}$$ (2)

$$\begin{array}{c} Constraint:y_{k}=w^{T}\phi \left(x\right)+b+e_{k}\;,\;k=1,2,\ldots ,n \end{array}$$ (3)

where e is the n × 1 error vector and λ is the regularization parameter which penalizes the error. From Eq 2, a Langrangian is formed, which is given as follows:

$$\begin{array}{c} \begin{array}{cc} L_{\left\{w,b,e,\alpha \right\}} & =J_{\left\{w,b,e\right\}}-\sum_{k=1}^{n}\alpha_{i}\left\{w^{T}\phi \left(x_{i}\right)+b+e_{k}-y_{k}\right\}\\ & =\frac{1}{2}w^{T}w+\frac{1}{2}\lambda \sum_{k=1}^{n}e_{k}^{2}-\sum_{k=1}^{n}\alpha_{i}\left\{w^{T}\phi \left(x_{i}\right)+b+e_{k}-y_{k}\right\} \end{array} \end{array}$$ (4)

Here, *α* is a Langrangian multiplier. Differentiating with respect to w, b, e, *α*, we get the conditions for optimality as:

$$\begin{array}{c} \left\{ \begin{array}{cc} & \frac{\partial L}{\partial w}=0\Rightarrow w=\sum_{k=1}^{n}\alpha_{i}\phi \left(x_{i}\right),\\ & \frac{\partial L}{\partial b}=0\Rightarrow \sum_{k=1}^{n}\alpha_{i}=0,\\ & \frac{\partial L}{\partial e}=0\Rightarrow \alpha_{k}=\lambda e_{k}\;,\;k=1,2,\ldots ,n\\ & \frac{\partial L}{\partial \alpha }=0\Rightarrow y_{k}=w^{T}\phi \left(x\right)+b+e_{k}\;,\;k=1,2,\ldots ,n \end{array}\right. \end{array}$$ (5)

Eliminating w and e, we get a system of linear equations as follows:

$$\begin{array}{c} \left[\begin{array}{cc} 0 & 1_{n}^{T}\\ 1_{n} & \Omega +\gamma I_{n} \end{array}][\begin{array}{c} b\\ \alpha \end{array}]=[\begin{array}{c} 0\\ Y \end{array}\right] \end{array}$$ (6)

where *Y* = [*y*_1_, …, *y*_*n*_]^*T*^, 1_*n*_ = [1, …, 1]^*T*^, *I*_*n*_ is an *n* × *n* identity matrix, *γ* = λ^−1^ is the hyperparameter and Ω ∈ *R*^*n*×*n*^ is the kernel matrix defined by Ω_*ij*_ = *ϕ*(*x*_*i*_)^*T*^ *ϕ*(*x*_*j*_) = *K*(*x*_*i*_, *x*_*j*_). *α* and b are the least square solutions to Eq 6. The LS-SVM regressor output is obtained as:

$$\begin{array}{c} y\left(x\right)=\sum_{k=1}^{n}\alpha_{i}K\left(x_{k},x\right)+b \end{array}$$ (7)

Several types of kernel functions can be used. They are as follows:

- Linear kernel: $K\left(x_{k},x\right)=x_{k}^{T}x$
- Polynomial kernel of degree d: $K\left(x_{k},x\right)={\left(1+x_{k}^{T}x/c\right)}^{d}$
- Sigmoid kernel: $K\left(x_{i},x\right)=tanh\left(cx_{k}^{T}x+\theta \right)$
- Radial basis function (RBF) kernel: *K*(*x*_*k*_, *x*) = *exp*(−‖*x* − *x*_*k*_‖^2^/2*σ*^2^)

Each type of kernel function has its own advantages. The linear kernel is faster and is usually used to express the linear component of the mapping relation, while the polynomial kernel has effective generalization and approximation abilities. The sigmoid kernel function is similar to a two-layer perceptron model, and is usually preferred for neural networks. In this work, we have used the RBF kernel as it has a wider convergence domain and an excellent learning ability in non-linear data without any prior knowledge.

### 3.2 Dragonfly algorithm

Dragonflies are a species of flying insects belonging to the order Odonata. They feed on small insects like mosquitoes and butterflies, and avoid predators like birds and spiders. They tend to exhibit two very interesting swarming behavioral aspects—static or feeding swarms, and dynamic or migratory swarms. In case of the former [44], they are usually seen forming groups of small sizes and flying over an area to look for possible food sources. For the latter case, dynamic swarms migrate away to a better food location [45]. DA [46] is inspired by these two types of behaviors which resemble the exploitation and exploration stages of a meta-heuristic based optimization algorithm, respectively. The mathematical description of the swarming behavior is controlled through five main parameters [47] which are described below, considering *N* as the total number of neighboring dragonflies:

- **Cohesion**: This is a parameter that controls the tendency of the dragonflies to move towards the center of mass (COM) of that specific neighborhood. It is calculated by the following equation:
  $$\begin{array}{c} C_{i}=\frac{\sum_{j=1}^{N}X_{j}}{N}-X \end{array}$$ (8)
  Here *X*_*j*_ is the position of the *j*^*th*^ neighboring dragonfly and *X* is the location of the current dragonfly.
- **Alignment**: This parameter gives an idea of the velocity of the current dragonfly with respect to the other neighboring dragonflies. It is mathematically expressed through the following equation:
  $$\begin{array}{c} A_{i}=\frac{\sum_{j=1}^{N}V_{j}}{N} \end{array}$$ (9)
  Here *V*_*j*_ stands for the velocity of the *j*^*th*^ neighboring dragonfly.
- **Separation**: As the name suggests, this parameter gives a mathematical idea of the separation between the dragonflies. This is reflected by the tendency of the swarm to avoid static collisions by taking each other’s separation into account. It is expressed through the following equation:
  $$\begin{array}{c} S_{i}=-\sum_{j=1}^{N}X-X_{j} \end{array}$$ (10)
  Here, *X*_*j*_ stands for the location of the *j*^*th*^ neighbor and *X* stands for the location of the current dragonfly.
- **Distraction**: This parameter controls how far away the dragonflies should fly to avoid the enemy. This is calculated as follows:
  $$\begin{array}{c} E_{i}=X+X^{-} \end{array}$$ (11)
  Here *X*^−^ is the enemy position and *X* is the position of the current dragonfly.
  This *EnemyPos* is a special variable that is calculated as the worst solution(Position of the dragonflies) found so far.
- **Attraction**: This parameter signifies how close to the food the dragonflies should come based on the current food location. It is expressed as follows:
  $$\begin{array}{c} F_{i}=X^{+}-X \end{array}$$ (12)
  Here *X*^+^ is the position of the food source and *X* is the position of the current dragonfly.
  This *FoodPos* is another special variable that is calculated as the best solution(Position of the dragonflies) found so far.

Another parameter called step vector (Δ*X*_*j*_) is introduced to calculate the next position of the dragonflies. This parameter is calculated with the help of the five previously mentioned parameters and some random constants. The step vector is calculated with the help of this equation:

$$\begin{array}{c} \Delta X_{t+1}=\left(sS_{i}+aA_{i}+cC_{i}+fF_{i}+eE_{i}\right)+w\Delta X_{t} \end{array}$$ (13)

Here the step vector of the *t*^*th*^ iteration is used for calculation of the step vector of the *t* + 1^*th*^ iteration. The five random weight coefficients *s*, *a*, *c*, *e*, *f* are multiplied with their respective parameters before adding them to the step vector.

Now for the calculation of the new position of the dragonflies, this step vector is added to the previous position.

$$\begin{array}{c} X_{t+1}=X_{t}+\Delta X_{t+1} \end{array}$$ (14)

One thing to note here is that, the neighboring region is defined as a *p*-dimensional (*p* is the number of dimensions in the optimization problem) space where the distance between two dragonflies in any dimension is not more than a radius value *r*. This *r* increases proportionally with the number of iterations passed, implying the neighboring region becomes bigger. Due to this behavior, the isolated dragonflies of initial smaller neighborhoods tend to explore more. As number of iteration increases, the increase in neighborhood size merges the previously smaller neighborhoods, and creates a bigger swarm of dragonflies. Then that bigger swarm tends to exploit the best solution up to that point. In smaller swarms (first stage of the whole process), the dragonflies have smaller alignment factor and higher cohesion factor values which help them to explore more. The reverse happens in case of exploitation (the second stage of the whole process). The transition from one phase to the other is done by changing the *r* value accordingly.

The convergence of the algorithm is guaranteed because when the exploration phase turns into exploitation, a dragonfly tends to see more dragonflies in its region and collectively moves away from the enemy, towards the food. (To support this idea, the worst solution is chosen as the enemy location and the best solution is chosen as the food). This behavior causes convergence towards a better solution and divergence from the non-promising solutions.

To increase the randomness of the dragonfly positions and to bring variety in search locations, the idea of Levy flight (a type of random walk) is introduced. When there is no other dragonfly in the neighborhood of a dragonfly, we use this idea to randomly place a dragonfly somewhere around its position. This Levy is calculated using the following equations:

$$\begin{array}{c} Levy\left(x\right)=0.01\times \frac{r_{1}\times \sigma }{r_{2}^{\frac{1}{\beta }}} \end{array}$$ (15)

$$\begin{array}{c} \sigma =(\frac{\Gamma \left(1+\beta \right)\times \text{sin}\left(\frac{\pi \beta }{2}\right)}{\Gamma \left(\frac{1+\beta }{2}\right)\times \beta \times 2^{\frac{\beta -1}{2}}}{)}^{\frac{1}{\beta }} \end{array}$$ (16)

Here Γ(*x*) = (*x* − 1)!.

Then we can update the position, using this following equation:

$$\begin{array}{c} X_{t+1}=Levy\left(d\right)\times X_{t}+X_{t} \end{array}$$ (17)

Here *d* is the dimension of the problem, *r*_1_ and *r*_2_ are two random numbers in the range [0, 1], and *β* is a constant taken as 1.5.

We also discuss some pros and cons of DA. The dragonflies start as small groups in their own regions which can be initialized to be far apart from one another, so they can do a lot of exploration which leads to finding varied solutions. In case of no neighbours, the dragonflies are spawned using Levy which adds required randomness to the search process, which further improves the exploration ability. The idea of moving away from the enemy and towards the food source makes sure that the dragonflies are searching for viable solutions. This idea paired with some modifications like altruism can lead to very efficient search of the space, which adds an edge to this algorithm. On the other hand, the dragonflies that once reach the boundaries have a tendency to stay at the boundaries, thereby reducing the effective number of dragonflies running the optimization process which can sometimes lead to poor results. In Dragonfly algorithm, there is a lot of neighbour calculation and updation steps, so adding a computationally heavy local search here would further increase the execution time of the algorithm.

### 3.3 Altruism

Altruism is a behavior that benefits others in spite of a cost to oneself. Its is motivated by selflessness and aims to increase the welfare of the community. A good example would be altruism among friends and family members. This behavior is observed in nature as well. In biological terms [48], the cost or benefit of altruism is determined on the basis of reproductive fitness, i.e, the expected number of children or offspring. So, the individual performing altruism reduces its chances of having a child but enhances the possibility of beneficiaries to have more children. In the proposed model, the concept of altruism has been incorporated in DA as described in subsection 4.2.

Altruism has some advantages which are worth mentioning. Firstly, it is a simple method that acts as a local search for the optimizer and it decreases the possibility of getting stuck in a local optimum. Moreover, it does not increase the computational cost too much as altruism itself takes very less time to execute. Based on social behavior, it is easy to conceptualize and implement. However, there is one main drawback of this method. There is no guarantee that altruism will improve the exploitation ability of the optimizer. This is because the beneficiary might not be that important. Since LS-SVM itself takes time to produce results, an efficient local search with low execution time is very important. A major point for including altruism in this study is that it has hitherto never been used in this domain, to the authors’ best knowledge. Keeping these facts in mind, we chose to incorporate altruism to the Dragonfly algorithm.

## 4 Proposed model

### 4.1 Preprocessing

Five technical indicators are calculated from the raw stock datasets:

- Relative Strength-Index (RS-Idx): It is a metric that measures the velocity and magnitude of price movements. The overbought and oversold conditions of an asset are found out using the ratio of recent gains to recent losses. It is calculated as follows:
  $$\begin{array}{c} Relative\;Strength\;\left(RS\right)=\frac{Upward\;price\;change}{Downward\;price\;change} \end{array}$$ (18)
  $$\begin{array}{c} RS-Idx=100-\frac{100}{1+RS} \end{array}$$ (19)
- M-Flow Index (MF-Idx): The volume of money in and out of a security is calculated using this indicator. Unlike RS-Idx which takes the price into account, M-Flow Index considers volume. Its value is derived as follows:
  $$\begin{array}{c} Typical\;Price=\frac{High+Low+Close}{3} \end{array}$$ (20)
  $$\begin{array}{c} M-Flow=Volume\times Typical\;Price \end{array}$$ (21)
  $$\begin{array}{c} M-Ratio=\frac{Positive\;M-Flow}{Negative\;M-Flow} \end{array}$$ (22)
  $$\begin{array}{c} M-Flow\;Index=100-\frac{100}{1+M-Ratio} \end{array}$$ (23)
- Exponential M-Average (EM-Avg): This metric gives the exponential moving average of a field over a given period of time. It is influenced significantly by most recent data points. EM-Avg formula is as follows:
  $$\begin{array}{c} EM-Avg=k\times TC+Y\times (1-k) \end{array}$$ (24)
  where TC = Today’s Closing price, Y = Yesterday’s EM-Avg and k = 2/(N+1). Here N is the number of days in EM-Avg.
- M-Average Convergence Divergence (M-ACD): M-ACD is a momentum indicator which denotes the relationship between the long-term and short-term moving averages of a security’s price. The formulae for calculating M-ACD and its signal as follows.
  $$\begin{array}{c} M-ACD=EM-Avg_{long}\times 0.075-EM-Avg_{short}\times 0.15 \end{array}$$ (25)
  $$\begin{array}{c} Signal\;Line=EM-Avg\;of\;M-ACD\times 0.2 \end{array}$$ (26)
  where *EM*-*Avg*_*long*_ and *EM*-*Avg*_*short*_ are the long-term and short-term EM-Avg of closing prices respectively.
- Stochastic Oscillator (SO): A stochastic oscillator is a momentum indicator comparing a particular closing price of a security to a range of its prices over a given duration of time. Its formula is as follows:
  $$\begin{array}{c} \%K=\frac{CP-LP}{HP-LP}\times 100 \end{array}$$ (27)
  where the most recent closing price is represented by CP, and the lowest price and highest price traded during the look-back period are denoted by LP and HP respectively. %K is the present value of the SO.

### 4.2 Altruistic Dragonfly algorithm

**Algorithm 1** Dragonfly Algorithm

Input: PopSize, dim, objFunc, bounds, maxIt

Output: bestFitness, bestLoc = (*x*_1_, *x*_2_, *x*_3_…., *x*_*d*_*im*)

 Initialize foodPos, enemyPos, X and Δ*X* randomly

 Initialize foodFitness, enemyFitness by running objFunc for foodPos and enemyPos respectively

 **for** *iter* ← 1 to *maxIt* **do**

  randomly assign values to *s*, *c*, *a*, *e*, *f*

  increase *r* in proportion to the *iter*

  calculate fitness value for all dragonflies and update enemyPos, enemyFitness, foodPos, and foodFitness.

  **for** *i* ← 1 to *PopSize* **do**

   Calculate the neighbors of the i-th dragonfly as described in 3.2

   Calculate the *S*, *C*, *A*, *E*, *F* values with Eqs (8) to (12)

   **if** number of neighbors is zero **then**

    update the position with Eq (17)

   **else**

    update the position and step vector using Eqs (13) and (14)

   **end if**

   check for out-of-bound values and bring them in range

  **end for**

 **end for**

 bestFitness = foodFitness

 bestLoc = foodPos

 return bestFitness and bestLoc

**Algorithm 2** Altruism in ADA

Choose the worst performing dragonfly as the altruist

Find all neighbors of the altruist dragonfly

Randomly choose one of its neighbors as the beneficiary

$rel=1-\frac{Distance\;between\;altruist\;and\;beneficiary}{Distance\;between\;altruist\;and\;its\;farthest\;neighbor}$

Move the altruist away from the food source and towards enemy; opposite for beneficiary

Ensure that all values are within prescribed bounds

Calculate the benefit for the beneficiary and the loss (cost) for the altruist

Check if Hamilton’s rule (Eq 28) is satisfied

If it is not satisfied, then abort the process and revert positions of altruist and beneficiary back to their original values

In this work, ADA has been proposed to find the most optimal combination of parameters of LS-SVM for stock price prediction. Two parameters are to be optimized: *γ*, which is the regularization parameter of LS-SVM, and *σ*, which is the kernel parameter. The position of a dragonfly represents the values *γ* and *σ*. The values of *γ* and *σ* are optimized using the equations described in subsection 3.2. At first, we have randomly initialized the positions of the dragonflies and then updated them using the best and worst solutions. The mean square error (MSE) between the actual stock prices and the predicted stock prices is used as the fitness function to judge the quality of the solutions. In the initial stage of the algorithm, the dragonflies explore various regions and find viable solutions, and in the later stages, they all start to group together and move towards the best solution found till then.

Altruism has been added while changing the position of dragonflies during exploration and exploitation. The dragonfly which performs altruism is known as the altruist and the dragonfly which receives the benefits is called the beneficiary. With respect to Eqs 11 and 12, the altruist moves away from the food source and towards the enemy. The opposite happens for the beneficiary, i.e., it moves towards the food source and away from the enemy. The positions of the altruist and the beneficiary are changed by a random number but they are always ensured to be between the lower and upper bounds of the problem. Altruism is carried out if Hamilton’s rule [49] is satisfied, according to Eq 28:

$$\begin{array}{c} rel\times ben>cost \end{array}$$ (28)

In the above equation, *ben* is the cost benefit or the reduction in fitness of the beneficiary, *cost* is the increase in fitness value of the altruist and *rel* is the relatedness or the relationship factor between the altruist and the beneficiary, i.e., how much related they are. In case this equation is not satisfied during altruism, or if the benefit of the recipient is less than zero, then altruism is not performed and the agent vectors revert back to their original values. Here the exploitation behavior comes into play. Finally after completing the maximum number of iterations, the dragonfly with the best fitness value gives the best values of *γ* and *σ*.

DA has been used recently in various fields of research like feature selection [50, 51]. Being a swarm-inspired algorithm, it also has the benefits of the well-known PSO. This algorithm has hitherto not been used for prediction of stock market prices. Moreover, the existence of an insect like dragonfly makes it ideal to incorporate the concept of altruism. The resultant performance is shown in the following sections.

**Fig 2:**
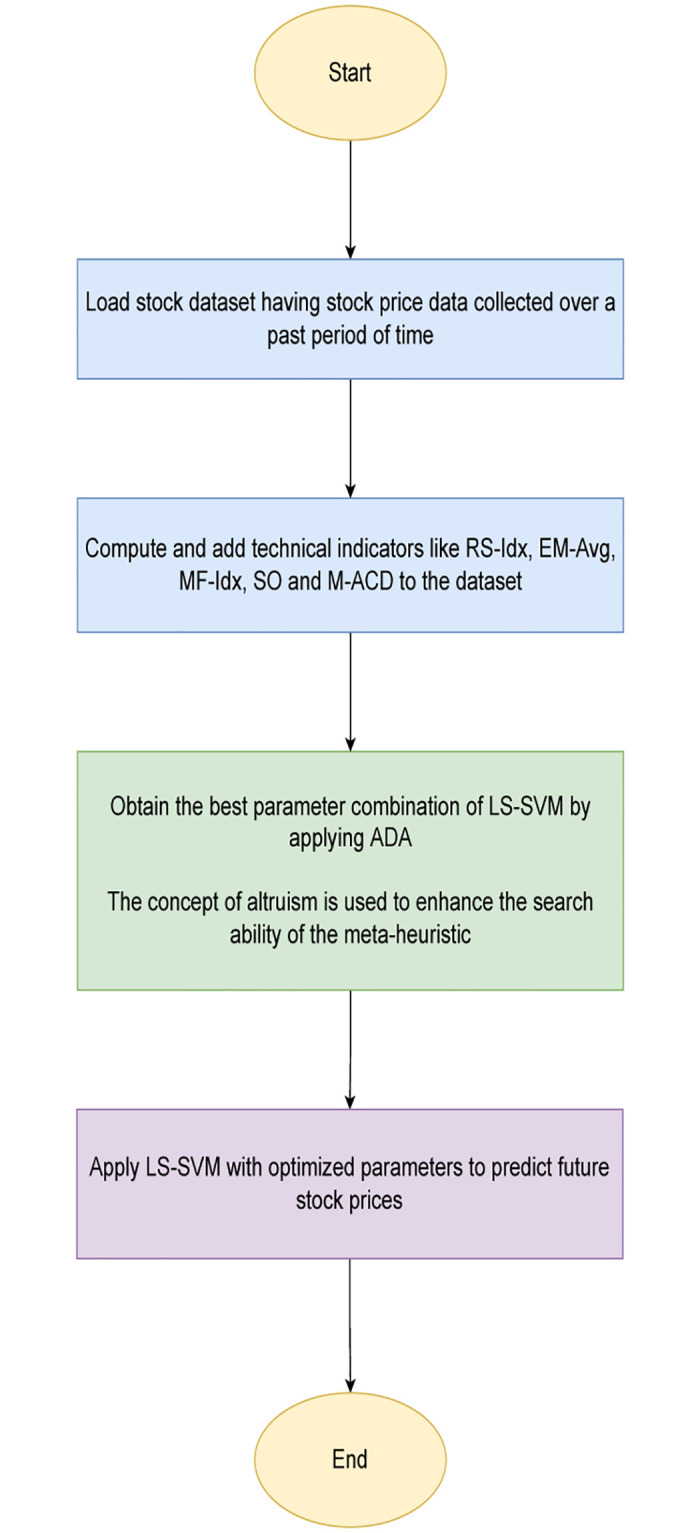
Flowchart describing the proposed model for predicting stock prices using LS-SVM optimized by ADA.

Fig 2 depicts the different steps involves the proposed model.

### 4.3 Time complexity analysis

The algorithm runs for *maxIt* times and for each run, it does the following things: Calculate fitness values for each agent using the objective function that requires one execution of the LSSVM and calculate the *EnemyPos*, *EnemyFitness*, *FoodPos*, *FoodFitness* values. *O*(*T*) for the objective function. Therefore, *O*(*n* * *T*) is the complexity of this step.

For each agent, decide the agents that are neighbours depending on the value of r and calculate the *s*, *c*, *a*, *f*, *e* values. Then update the position accordingly. *O*(*n*) is the complexity for one agent’s neighbour calculation. Therefore, *O*(*n*^2^) is the complexity of this step.

Apply altruism on the new position values. Calculate neighbors of altruist and choose one. Then perform altruism and calculate benefit and loss. *O*(*n*) is the complexity for altruist’s neighbour calculation, and *O*(*T*) is the complexity for loss and benefit calculations. Therefore, *O*(*n* + *T*) is the complexity of this step.

The time complexity of the algorithm is found to be *O*((*n*^2^ + *n* * *T*) * *maxIt*). Here, *n* is the population size of the ADA, and *maxIt* is the total number of iterations. *T* is the complexity of the objective function requiring one execution of LSSVM, which is typically quadratic in the size of the training dataset.

## 5 Experiments

This section deals with the experiments performed. For each company, stock prices from January 2017 to October 2021 are considered, with the first 900 days taken as training set and the next 290 days taken as the testing set. The proposed model is implemented using Python3 [52]. The experiments are conducted on a PC with 3.30 GHz Intel(R) Pentium(R) CPU G4400 and 8 GB Memory.

### 5.1 Parameter tuning

The three main parameters of DA have been given in Table 1. The *PopSize* i.e., population size is tested for [10, 20, 30, 50] and finally it is set as 20 as it gives the best results. Another attribute *β* has been set following the original paper on DA. Apart from these, the others parameters like *s*, *c*, *a*, *f*, *e* are calculated based on the current number of iterations. These parameters help the algorithm to switch from exploration to exploitation and ensures that the search space of the dragonflies are searched thoroughly.

**Table 1:** Parameter values of DA.

Parameter	Description	Value
maxIt	total iterations	100
PopSize	number of dragonflies	20
*β*	attribute of Levy	1.5

### 5.2 Results on stock market datasets

For experimental purposes, we have used seven well-known algorithms of Whale Optimization Algorithm (WOA) [53], Salp Swarm Algorithm (SSA) [54], PSO, GSA, GA, Sine Cosine Algorithm (SCA) [55] and Dragonfly Algorithm, apart from the proposed method ADA. They are chosen because all the seven methods are quite popular and have been previously used in the finance domain as well as for other purposes such as feature selection. Each of the eight algorithms is combined with LS-SVM and tested on the the publicly available datasets of 12 companies. The values in Table 2 denote the MSE of the respective algorithm on a dataset. The last column represents the MSE for the proposed model.

**Table 2:** Values of the fitness function for different algorithms run on various companies’ datasets.

Company	WOA	SSA	PSO	GSA	GA	SCA	DA	ADA
Adobe	0.2078	0.2067	0.1968	0.2075	0.2015	0.1994	0.1993	**0.1863**
American Express	0.2218	0.2388	0.2219	**0.2207**	0.2246	0.2242	0.2252	0.2216
Apple	0.1276	0.1479	0.1323	0.1356	0.1244	0.1227	0.1288	**0.1220**
AT&T	0.2473	0.2489	0.2465	0.2465	0.2283	0.2271	0.2272	**0.2160**
Bank of New York	0.2901	**0.2798**	0.3103	0.3024	0.2928	0.2929	0.2929	**0.2798**
Coca-Cola	0.2848	0.2870	0.2856	0.2820	0.2519	0.2462	0.2461	**0.2428**
ExxonMobil	0.1716	0.1697	0.1770	**0.1601**	0.1686	0.1647	0.1647	0.1607
FMC	0.2673	0.2661	0.2577	0.2560	0.2661	0.2651	0.2457	**0.2420**
HP	0.2420	0.2058	0.2408	0.2394	0.2940	0.2954	0.2845	**0.2053**
Honeywell	0.1921	**0.1844**	0.1967	0.1898	0.2002	0.1970	0.1970	0.1904
Oracle	0.2820	0.2794	0.2819	0.2804	0.2717	0.2710	0.2710	**0.2656**
Tesla	0.1621	0.1597	**0.1452**	0.1634	0.1668	0.1578	0.1578	0.1466
**Average Rank**	5.25	5	4.75	4.16	4.91	3.91	3.83	**1.41**
**Assigned Rank**	8	7	5	4	6	3	2	**1**

Our aim would be to minimize the MSE. Analyzing Table 2, we can see that the proposed method gives the best results for 8 out of 12 datasets; the only exceptions are American Express, ExxonMobil, Honeywell and Tesla. Moreover, there is a tie between SSO and the proposed method in case of Bank of New York. In case of American Express, ExxonMobil and Tesla, the difference between the MSE’s of ADA and the respective best algorithm is even lesser than 1%. So the proposed work is almost as good as the best method in case of these 3 datasets; the only significant difference is in case of Honeywell where the MSE of ADA deviates by 3.25%.

Next, we compare the result obtained by ADA with respect to naive Dragonfly algorithm. The proposed model based on ADA gives better results than the original Dragonfly algorithm for all the 12 datasets considered, and that too by a significant margin. The difference in their MSE’s is particularly stark for HP, differing by almost 0.08, i.e., by 28%. So we can extrapolate that altruism adds value to original algorithm and improves the overall efficiency of the algorithm.

The original Dragonfly algorithm is the best amongst all the methods except ADA. Thus, it is a good choice for stock market prediction in itself. ADA adds even more value to it. The average rank for the proposed method is the least amongst the eight algorithms so it is assigned the first rank. The proposed model displays a very high level of prediction accuracy since MSE is comparatively low across most of the datasets. Even for datasets like American Express, ExxonMobil and Tesla, where it fails to produce the best results, its results are not significantly worse than those of the best performing algorithm. Thus, ADA combined with LS-SVM can be considered as a state-of-the-art algorithm in this domain. We can infer that the model proposed in this work can be reliably used for predicting the future stock prices of companies.

**Fig 3:**
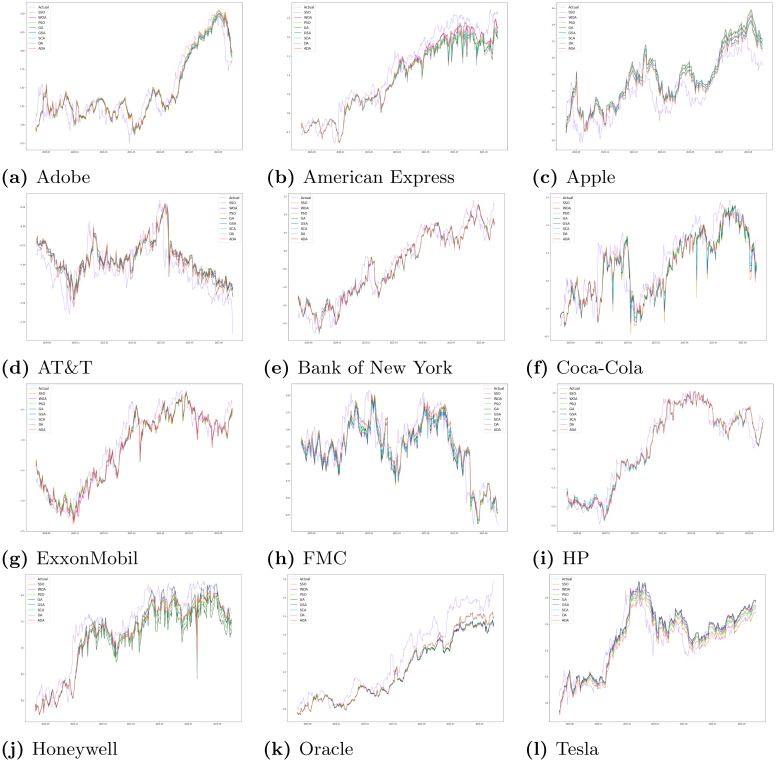
Predicted stock prices of 12 companies using 8 different algorithms. (a) Adobe, (b) American Express, (c) Apple, (d) AT&T, (e) Bank of New York, (f) Coca-Cola, (g) ExxonMobil, (h) FMC, (i) HP, (j) Honeywell, (k) Oracle, (l) Tesla.

Fig 3 shows the forecast on the data of 12 companies by all the methods taken for comparison purposes. The period shown in the graphs are approximately from September 2020 till September 2021, equal to 290 days. This particular period was chosen as it reflects the changing stock prices during the COVID-19 induced economic recession and later on, a boom. For Fig 3, the other optimization methods as well as ADA are combined with LS-SVM. We can see that the proposed model gives the most accurate forecasts and shows a high level of precision and forecasting accuracy. ADA is able to accurately predict and follow the trend in the stock prices of companies, even during the COVID period. The results are vindicated by Table 2 as well. Therefore, the proposed method is very reliable for forecasting purposes. In Table 3, the MSE values for ADA with Linear Kernel LS-SVM on different datasets are given. We can see that our proposed model with RBF Kernel LS-SVM produces significantly better results than the one using Linear Kernel.

**Table 3:** Values of the fitness function for linear kernel LS-SVM algorithm run on various companies’ datasets.

Company	Fitness
Adobe	0.2257
American Express	0.3302
Apple	0.3471
AT&T	0.2632
Bank of New York	0.2600
Coca-Cola	0.4060
ExxonMobil	0.1913
FMC	0.2057
HP	0.1525
Honeywell	0.3814
Oracle	0.2945
Tesla	0.1627

### 5.3 Results on benchmark functions

Two categories of test functions [56] have been used to benchmark the performance of the proposed ADA with respect to the other meta-heuristic algorithms, and the results are given in Table 4. The first category includes unimodal functions (F1-F5) that have a single optimum solution, so these are used to test the exploitation ability and convergence of the algorithm. The second category includes multi-modal functions (F8-F11) that have more than one local optimum and one global optimum. Thus, the algorithm needs to be able to globally search the space and avoid being trapped in local optima in order to find the global optimum.

**Table 4:** Optimization results of the 8 different algorithms including ADA on 12 benchmark functions.

Test Function		WOA	SSA	PSO	GSA	GA	SCA	DA	ADA
F1	Ave	0.0000	0.0000	0.0003	0.0000	748.5972	0.0000	0.0000	0.0000
	Std	0.0000	0.0000	0.0002	0.0000	324.9262	0.0000	0.0000	0.0000
F2	Ave	0.0000	0.2272	0.0421	0.0557	5.9714	0.0000	0.0000	0.0000
	Std	0.0000	1.0000	0.0454	0.1941	1.5331	0.0001	0.0000	0.0000
F3	Ave	0.0000	0.0000	70.1256	896.5347	1949.003	0.0371	0.0000	0.0000
	Std	0.0000	0.0000	22.1192	318.956	994.2733	0.1372	0.0028	0.0004
F4	Ave	0.0725	0.0000	1.0844	7.3549	21.1630	0.0965	0.0009	0.0000
	Std	0.3975	0.6556	0.3171	1.7414	2.6054	0.5823	0.0028	0.0135
F5	Ave	27.8658	5.0478	96.7183	67.5431	133307.1	0.0009	28.7605	26.7712
	Std	0.7636	2.0612	60.1156	62.2253	85007.6	0.0017	6.7865	3.5614
F8	Ave	−5080.76	-8896.21	−4841.29	−2821.07	-3407.25	-10572.34	-8282.39	-10212.76
	Std	695.796	878.293	1152.82	493.037	164.478	618.452	383.6466	514.729
F9	Ave	0.0000	0.4254	46.7042	25.9684	25.5189	0.0000	0.0000	0.0000
	Std	0.0000	0.9502	11.6294	7.4701	6.6694	0.7303	9.4791	2.4735
F10	Ave	7.4043	0.0598	0.2760	0.0621	9.4988	0.3804	0.0000	0.0000
	Std	9.8975	0.5279	0.5090	0.2363	1.2714	1.0000	0.4871	0.0005
F11	Ave	0.0002	0.0000	0.0092	27.7015	7.7200	0.0000	0.0000	0.0000
	Std	0.0016	0.0000	0.0077	5.0403	3.6261	0.0051	0.0735	0.0566
F12	Ave	0.3397	0.2251	0.0069	1.7996	1858.51	0.0000	0.1909	0.1543
	Std	0.2148	0.0000	0.0263	0.9511	5820.215	0.0000	0.0983	0.0247

### 5.4 Statistical significance test

To assess the statistical significance of the results obtained in Table 2, Wilcoxon rank-sum test is used. It is a non-parametric statistical test where pairwise comparison is done [57]. The null hypothesis is that two sets of results have the same distribution. However, if they are statistically different, then the generated probability value or the *p*-value will be less than the significance level of 0.05. Thereafter, the null hypothesis will be rejected.

Looking at the results of Tables 5 and 6, we can infer that all the results are statistically significant as all the *p*-values are less than 0.05 in each of these tables.

**Table 5:** *p*-values of the Wilcoxon rank-sum test in terms of MSE of the proposed method on stock market datasets.

	WOA	SSA	PSO	GSA	GA	SCA	DA
**ADA**	0.002	0.026	0.027	0.013	0.031	0.011	0.028

**Table 6:** *p*-values of the Wilcoxon rank-sum test in terms of average of benchmark functions.

	WOA	SSA	PSO	GSA	GA	SCA	DA
**ADA**	0.04	0.034	0.02	0.001	0.005	0.03	0.026

## 6 Data and code availability

All of the 12 datasets used in this paper are available for free over the internet and can be consumed with the following API: https://finance.yahoo.com/quotes/API,Documentation/view/v1/. Moreover, the whole datasets and the working code are uploaded at the GitHub repository: https://github.com/SayanAcharya2002/StockMarketPrediction.

## 7 Conclusion and future work

In this paper, we have proposed a machine learning model using LS-SVM with its parameters optimized using the meta-heuristic ADA and applied it to predict future stock prices. Optimal values of the regularization parameter *γ* and kernel parameter *σ* have been found out using the modified version of DA. The proposed model has been applied on stock price datasets of 12 companies namely Adobe, American Express, Apple, AT&T, Bank of New York, Coca-Cola, ExxonMobil, FMC, HP, Honeywell, Oracle and Tesla. The results obtained from the proposed model are compared with those obtained by using seven other meta-heuristic optimization algorithms. Analysis of the results show that the proposed model performs well and has the ability to deal with the irregularities and fluctuations associated with stock price data. For future work, different initialization strategies can be used with ADA to improve population diversity. Depending on the nature of the work, ADA can be used with regression models other than LS-SVM. Local search techniques may be added to further improve exploitation ability. We can apply the proposed model on other types of time series data like gasoline price, unemployment rate in a state, amount of sales, etc. and check if is able to produce reliable forecasting results.

## References

[1] Ariyo AA, Adewumi AO, Ayo CK. Stock price prediction using the ARIMA model. In: 2014 UKSim-AMSS 16th International Conference on Computer Modelling and Simulation. IEEE; 2014. p. 106–112.

[2] Dase R, Pawar D. Application of Artificial Neural Network for stock market predictions: A review of literature. International Journal of Machine Intelligence. 2010;2(2):14–17. doi: 10.9735/0975-2927.2.2.14-17

[3] Cortes C, Vapnik V. Support-vector networks. Machine Learning. 1995;20(3):273–297. doi: 10.1007/BF00994018

[4] Cherkassky V, Ma Y. Practical selection of SVM parameters and noise estimation for SVM regression. Neural networks. 2004;17(1):113–126. doi: 10.1016/S0893-6080(03)00169-2 14690712

[5] Suykens JA, Vandewalle J. Least squares support vector machine classifiers. Neural processing letters. 1999;9(3):293–300. doi: 10.1023/A:1018628609742

[6] Alvarez Meza AM, Daza Santacoloma G, Acosta Medina CD, Castellanos Dominguez G. Parameter selection in least squares-support vector machines regression oriented, using generalized cross-validation. Dyna. 2012;79(171):23–30.

[7] Goodell JW. COVID-19 and finance: Agendas for future research. Finance Research Letters. 2020;35:101512. doi: 10.1016/j.frl.2020.101512 32562472 PMC7152896

[8] Gupta M, Abdelmaksoud A, Jafferany M, Lotti T, Sadoughifar R, Goldust M. COVID-19 and economy. Dermatologic therapy. 2020;33(4):e13329–e13329. doi: 10.1111/dth.13329 32216130 PMC7228404

[9] Baker SR, Bloom N, Davis SJ, Kost K, Sammon M, Viratyosin T. The Unprecedented Stock Market Reaction to COVID-19. The Review of AssetPricing Studies. 2020;10(4):742–758.

[10] Mazur M, Dang M, Vega M. COVID-19 and the march 2020 stock market crash. Evidence from S&P1500. Finance Research Letters. 2021;38:101690. doi: 10.1016/j.frl.2020.101690 32837377 PMC7343658

[11] Nti IK, Adekoya AF, Weyori BA. A systematic review of fundamental and technical analysis of stock market predictions. Artificial Intelligence Review. 2019;53(4):3007–3057. doi: 10.1007/s10462-019-09754-z

[12] Chen JF, Do QH, Hsieh HN. Training artificial neural networks by a hybrid PSO-CS algorithm. Algorithms. 2015;8(2):292–308. doi: 10.3390/a8020292

[13] Kim HJ, Jo NO, Shin KS. Optimization of cluster-based evolutionary undersampling for the artificial neural networks in corporate bankruptcy prediction. Expert systems with applications. 2016;59:226–234. doi: 10.1016/j.eswa.2016.04.027

[14] de Menezes LHS, Carneiro LL, de Carvalho Tavares IM, Santos PH, das Chagas TP, Mendes AA, et al. Artificial neural network hybridized with a genetic algorithm for optimization of lipase production from Penicillium roqueforti ATCC 10110 in solid-state fermentation. Biocatalysis and Agricultural Biotechnology. 2021;31:101885. doi: 10.1016/j.bcab.2020.101885

[15] Arunchai T, Sonthipermpoon K, Apichayakul P, Tamee K. Resistance spot welding optimization based on artificial neural network. International Journal of Manufacturing Engineering. 2014;2014. doi: 10.1155/2014/154784

[16] Richards SM, Berkenkamp F, Krause A. The lyapunov neural network: Adaptive stability certification for safe learning of dynamical systems. In: Conference on Robot Learning. PMLR; 2018. p. 466–476.

[17] Chaoui H, Sicard P. Adaptive Lyapunov-based neural network sensorless control of permanent magnet synchronous machines. Neural Computing and Applications. 2011;20(5):717–727. doi: 10.1007/s00521-010-0412-6

[18] qiang Xie G. The Optimization of Share Price Prediction Model Based on Support Vector Machine. IEEE; 2011. Available from: 10.1109/iccase.2011.5997714.

[19] Qiu M, Song Y. Predicting the Direction of Stock Market Index Movement Using an Optimized Artificial Neural Network Model. PLOS ONE. 2016;11(5):e0155133. doi: 10.1371/journal.pone.0155133 27196055 PMC4873195

[20] Yu H, Liu H. Improved Stock Market Prediction by Combining Support Vector Machine and Empirical Mode Decomposition. IEEE; 2012. Available from: 10.1109/iscid.2012.138.

[21] Huang Y, Gao Y, Gan Y, Ye M. A new financial data forecasting model using genetic algorithm and long short-term memory network. Neurocomputing. 2021;425:207–218. doi: 10.1016/j.neucom.2020.04.086

[22] Huang C, li Huang L, ting Han T. Financial time series forecasting based on wavelet kernel support vector machine. IEEE; 2012. Available from: 10.1109/icnc.2012.6234569.

[23] Siedlecki W, Sklansky J. A Note On Genetic Algorithms for Large-Scale Feature Selection. In: Handbook of Pattern Recognition and Computer Vision. World Scientific; 1993. p. 88–107.

[24] Koza JR, Koza JR. Genetic programming: on the programming of computers by means of natural selection. vol. 1. MIT press; 1992.

[25] Kennedy J, Eberhart R. Particle swarm optimization. In: Proceedings of ICNN’95-International Conference on Neural Networks. vol. 4. IEEE; 1995. p. 1942–1948.

[26] Zervoudakis K, Tsafarakis S. A mayfly optimization algorithm. Computers & Industrial Engineering. 2020;145:106559. doi: 10.1016/j.cie.2020.106559

[27] Kirkpatrick S, Gelatt CD, Vecchi MP. Optimization by simulated annealing. science. 1983;220(4598):671–680. doi: 10.1126/science.220.4598.671 17813860

[28] Rashedi E, Nezamabadi-Pour H, Saryazdi S. GSA: a gravitational search algorithm. Information sciences. 2009;179(13):2232–2248. doi: 10.1016/j.ins.2009.03.004

[29] Geem ZW, Kim JH, Loganathan GV. A new heuristic optimization algorithm: harmony search. simulation. 2001;76(2):60–68. doi: 10.1177/003754970107600201

[30] Kashan AH. League championship algorithm: a new algorithm for numerical function optimization. In: 2009 international conference of soft computing and pattern recognition. IEEE; 2009. p. 43–48.

[31] Zhiqiang G, Huaiqing W, Quan L. Financial time series forecasting using LPP and SVM optimized by PSO. Soft Computing. 2013;17(5):805–818. doi: 10.1007/s00500-012-0953-y

[32] Thakkar A, Chaudhari K. A Comprehensive Survey on Portfolio Optimization, Stock Price and Trend Prediction Using Particle Swarm Optimization. Archives of Computational Methods in Engineering. 2020;28(4):2133–2164. doi: 10.1007/s11831-020-09448-8

[33] Hegazy O, Soliman OS, Salam MA. Lssvm-abc algorithm for stock price prediction. arXiv preprint arXiv:14026366. 2014;.

[34] Ghanbari M, Arian H. Forecasting stock market with support vector regression and butterfly optimization algorithm. arXiv preprint arXiv:190511462. 2019;.

[35] Nair BB, Mohandas V, Sakthivel N. A genetic algorithm optimized decision tree-SVM based stock market trend prediction system. International Journal on Computer Science and Engineering. 2010;2(9):2981–2988.

[36] Chandar SK. Grey Wolf optimization-Elman neural network model for stock price prediction. Soft Computing. 2020;25(1):649–658. doi: 10.1007/s00500-020-05174-2

[37] Du H. Implementation of Improved Fruit Fly Optimization Algorithm in Stock Market Segment Analysis and Forecasting. IEEE; 2019. Available from: 10.1109/icris.2019.00131

[38] Sands TM, Tayal D, Morris ME, Monteiro ST. Robust stock value prediction using support vector machines with particle swarm optimization. In: 2015 IEEE Congress on Evolutionary Computation (CEC). IEEE; 2015. Available from: 10.1109/cec.2015.7257306.

[39] Chung H, shik Shin K. Genetic algorithm-optimized multi-channel convolutional neural network for stock market prediction. Neural Computing and Applications. 2019;32(12):7897–7914. doi: 10.1007/s00521-019-04236-3

[40] Das SR, Mishra D, Rout M. Stock market prediction using Firefly algorithm with evolutionary framework optimized feature reduction for OSELM method. Expert Systems with Applications: X. 2019;4:100016. doi: 10.1016/j.eswax.2019.100016

[41] Seidy EE. A new particle swarm optimization based stock market prediction technique. International Journal of Advanced Computer Science and Applications. 2016;7(2).

[42] Hegazy O, Soliman OS, Salam MA. Comparative study between FPA, BA, MCS, ABC, and PSO algorithms in training and optimizing of LS-SVM for stock market prediction. International Journal of Advanced Computer Research. 2015;5(18):35–45.

[43] Vapnik V. The nature of statistical learning theory. Springer science & business media; 1999.

[44] Wikelski M, Moskowitz D, Adelman JS, Cochran J, Wilcove DS, May ML. Simple rules guide dragonfly migration. Biology Letters. 2006;2(3):325–329. doi: 10.1098/rsbl.2006.0487 17148394 PMC1686212

[45] Russell RW, May ML, Soltesz KL, Fitzpatrick JW. Massive swarm migrations of dragonflies (Odonata) in eastern North America. The American Midland Naturalist. 1998;140(2):325–342. doi: 10.1674/0003-0031(1998)140[0325:MSMODO]2.0.CO;2

[46] Mirjalili S. Dragonfly algorithm: a new meta-heuristic optimization technique for solving single-objective, discrete, and multi-objective problems. Neural Computing and Applications. 2016;27(4):1053–1073. doi: 10.1007/s00521-015-1920-1

[47] Reynolds CW. Flocks, herds and schools: A distributed behavioral model. In: Proceedings of the 14th annual conference on Computer graphics and interactive techniques; 1987. p. 25–34.

[48] Prudkov PN, Rodina ON. On altruism toward nonhuman animals. Society & Animals. 2016;24(4):321–336. doi: 10.1163/15685306-12341419

[49] Waibel M, Floreano D, Keller L. A Quantitative Test of Hamilton’s Rule for the Evolution of Altruism. 2011;9(5):e1000615. 10.1371/journal.pbio.1000615 PMC3086867 21559320

[50] Mafarja MM, Eleyan D, Jaber I, Hammouri A, Mirjalili S. Binary dragonfly algorithm for feature selection. In: 2017 International conference on new trends in computing sciences (ICTCS). IEEE; 2017. p. 12–17.

[51] Hammouri AI, Mafarja M, Al-Betar MA, Awadallah MA, Abu-Doush I. An improved dragonfly algorithm for feature selection. Knowledge-Based Systems. 2020;203:106131. doi: 10.1016/j.knosys.2020.106131

[52] van Rossum G, Drake FL. The Python Language Reference Manual. Network Theory Ltd.; 2011.

[53] Mirjalili S, Lewis A. The Whale Optimization Algorithm. Advances in Engineering Software. 2016;95:51–67. doi: 10.1016/j.advengsoft.2016.01.008

[54] Mirjalili S, Gandomi AH, Mirjalili SZ, Saremi S, Faris H, Mirjalili SM. Salp Swarm Algorithm: A bio-inspired optimizer for engineering design problems. Advances in Engineering Software. 2017;114:163–191. doi: 10.1016/j.advengsoft.2017.07.002

[55] Mirjalili S. SCA: A Sine Cosine Algorithm for solving optimization problems. Knowledge-Based Systems. 2016;96:120–133. doi: 10.1016/j.knosys.2015.12.022

[56] Yao X, Liu Y, Lin G. Evolutionary programming made faster. IEEE Transactions on Evolutionary Computation. 1999;3(2):82–102. doi: 10.1109/4235.771163

[57] Dhargupta S, Ghosh M, Mirjalili S, Sarkar R. Selective Opposition based Grey Wolf Optimization. Expert Systems with Applications. 2020; p. 113389. doi: 10.1016/j.eswa.2020.113389

